# H_2_O_2_ Inhibits ABA-Signaling Protein Phosphatase HAB1

**DOI:** 10.1371/journal.pone.0113643

**Published:** 2014-12-02

**Authors:** Madhuri Sridharamurthy, Amanda Kovach, Yang Zhao, Jian-Kang Zhu, H. Eric Xu, Kunchithapadam Swaminathan, Karsten Melcher

**Affiliations:** 1 Laboratories of Structural Sciences/Structural Biology and Biochemistry, Van Andel Research Institute, N.E., Grand Rapids, Michigan, United States of America; 2 Department of Biological Sciences, National University of Singapore, Singapore 117543, Singapore; 3 Department of Horticulture and Landscape Architecture, Purdue University, West Lafayette, Indiana 47906, United States of America; 4 State Key Laboratory of Drug Research, VARI-SIMM Center, Center for Structure and Function of Drug Targets, Shanghai Institute of Materia Medica, Shanghai Institutes for Biological Sciences, Chinese Academy of Sciences, Shanghai, People's Republic of China; Iowa State University, United States of America

## Abstract

Due to its ability to be rapidly generated and propagated over long distances, H_2_O_2_ is an important second messenger for biotic and abiotic stress signaling in plants. In response to low water potential and high salt concentrations sensed in the roots of plants, the stress hormone abscisic acid (ABA) activates NADPH oxidase to generate H_2_O_2_, which is propagated in guard cells in leaves to induce stomatal closure and prevent water loss from transpiration. Using a reconstituted system, we demonstrate that H_2_O_2_ reversibly prevents the protein phosphatase HAB1, a key component of the core ABA-signaling pathway, from inhibiting its main target in guard cells, SnRK2.6/OST1 kinase. We have identified HAB1 C186 and C274 as H_2_O_2_-sensitive thiols and demonstrate that their oxidation inhibits both HAB1 catalytic activity and its ability to physically associate with SnRK2.6 by formation of intermolecular dimers.

## Introduction

While long only considered as toxic byproducts of aerobic metabolism, reactive oxygen species (ROS) are now established as important second messengers in plants and animals [1], [2], [3]. In plants they play widespread roles in immunity, cell death, abiotic stress, and regulation of stomatal closure, and ROS production is induced by stress-related phytohormones, such as jasmonate, salicylic acid, ethylene, and abscisic acid [4], [5], [6], [7], [8]. The key reactive oxygen species for signaling is H_2_O_2_, which is generated by inducible NADPH oxidases, in combination with superoxide dismutase. Compared to other ROS, H_2_O_2_ is a relatively mild oxidant. Its ability to be rapidly generated and removed and to self-propagate over long distances makes H_2_O_2_, in spite of its toxicity, an ideal “early warning system” for both abiotic and biotic stress in plants [9], [10].

The hormone abscisic acid (ABA) plays a key role in sensing and adapting to abiotic stresses, such as drought, cold, and salinity [11], [12], [13], [14]. The central signaling module of the ABA pathway consists of three major components: PYR/PYL/RCAR ABA receptors, type 2C protein phosphatases (PP2Cs), and subclass 2 Snf1-related kinases (SnRK2s) [15], [16], [17], [18], [19]. In the absence of ABA, PP2Cs keep SnRK2s in an inactive state by dephosphorylating a critical serine residue in the activation loop and by forming physical complexes with SnRK2s that block substrate access [20], [21], [22]. Binding of ABA to the intracellular PYR/PYL/RCAR receptors induces a conformational change that allows them to bind and inactivate PP2Cs, thereby preventing inhibition of the SnRK2s and allowing the SnRK2s to phosphorylate their downstream targets [23], [24], [25].

Stomatal closure is an important mechanism adopted by plants to prevent water loss by transpiration during water-deficient conditions [26]. It is the earliest plant response to water stress and is largely driven by a complex interplay of proton pumps and ion channels to mediate a net ion efflux and shrinking of guard cells. These processes are regulated by ABA, whose level increases during water stress [27]. In *Arabidopsis* ABI1, ABI2, PP2CA, and HAB1 are the major PP2Cs involved in negative regulation of the ABA pathway during stomatal closure [28], [29], [30], [31], [32], [33], [34]. These PP2Cs share a highly conserved catalytic domain but they differ greatly in their N-terminal region [28]. SnRK2.6 (also known as OST1; Open stomata 1) is the key SnRK2 and positive regulator of the pathway in guard cells [14], [35], [36], [37], [38]. ABA-mediated inactivation of PP2Cs prevents the inhibition of SnRK2.6 to allow its auto-phosphorylation and auto-activation [39], [40], [41], [42], [43], [44]. Activated SnRK2.6 in turn phosphorylates and regulates the activity of various downstream effector proteins such as the guard cell ion channels KAT1 and SLAC1, NADPH oxidase AtrbohF, and the transcription factors ABF2, ABI5, and ABI4, thereby changing the protein and ion profile of the cell and leading to stomatal closure [45], [46], [47], [48].

Previous studies have shown that ABI1 and ABI2 are sensitive to H_2_O_2_ with reported phosphatase IC_50_ values of 140 and 50 µM, respectively [49], [50]. Moreover, ABA is unable to induce ROS production in *abi1-1*, but not *abi2-1*, mutants, indicating that PP2Cs and ROS have complex regulatory connections [51]. These findings led us to question whether HAB1 is also regulated by H_2_O_2_. Indeed, our results indicate that HAB1 is sensitive to H_2_O_2_. HAB1 lost its protein phosphatase activity with an IC_50_ of 340 µM when treated with H_2_O_2_. The two cysteine residues C186 and C274 act as redox sensing thiols in HAB1. Furthermore, H_2_O_2_-inactivated HAB1 cannot bind to SnRK2.6 and inhibit its kinase activity, resulting in activation of the ABA signaling pathway in a reconstituted in vitro system. These findings suggest that various protein phosphatases of the ABA signaling pathway are regulated by H_2_O_2_ and that ROS-mediated PP2C inhibition may serve as a feedback mechanism to amplify/enhance the downstream signaling of ABA-induced stomatal closure during water-deficient conditions.

## Materials and Methods

### Expression and purification of recombinant proteins

The *Arabidopsis thaliana* HAB1 phosphatase domain (171–511 aa) and C→S mutants were expressed as His_6_-GST fusion proteins in the expression vector pET24a (Novagen). Two liters of BL21 (DE3) cells were grown to an OD_600_ of 1.0, followed by induction with 100 µM of isopropyl-beta-D-thio-galactopyranoside (IPTG) at 16°C. For HAB1 cultures, 10 mM MgCl_2_ was added during induction. After overnight incubation, cells were harvested and resuspended in 100 ml of buffer A (20 mM Tris, pH 8.0, 200 mM NaCl, 10 mM MgCl_2_, 10% glycerol,) containing 2 mM β-mercaptoethanol, and 200 µl of saturated PMSF solution. Cells were lysed using a French Press. Lysates were centrifuged at 30,000 g for 30 min and the supernatant was loaded onto a 5 ml Ni-chelating Hi-Trap column (Amersham Biosciences) equilibrated with buffer A. The column was washed with 100 ml of Buffer A containing 20 mM imidazole and the fusion protein was eluted with Buffer A containing 500 mM imidazole. Eluted proteins were subjected to overnight thrombin incubation at 4°C to cleave the His_6_-GST tag and were passed through a second Ni column to separate the tag from the untagged proteins. Untagged proteins were further purified by Superdex 200 gel filtration chromatography (Amersham Biosciences) in Buffer A containing 1 mM dithiotreitol (DTT). Purified proteins were concentrated, aliquoted, and stored at −80°C. His_6_-GST-ABF2(73–120 aa), His_6_-GST-SnRK2.6(11–362 aa), His_6_-MBP-HAB1(1–511 aa), His_6_-MBP-HAB1(1–511 aa) C18S6S/C274S, His_6_-MBP-HAB1(171–511 aa), His_6_-MBP-HAB1(171–511 aa) C18S6S/C274S and His_6_-MBP-HAB1(171–511 aa) R505A were also purified similarly, but without the thrombin cleavage step. Nickel elutes were directly loaded onto a Superdex 200 gel filtration column.

To generate biotinylated proteins for luminescence proximity assays (AlphaScreen), SnRK2.6 (11–362 aa) and PYR1 (9–182 aa) open reading frames (orf) were cloned into a pETDuet (Novagen) derivative vector and were expressed in *E. coli* BL21 (DE3) cells. The first T7 polymerase-driven expression unit of this vector contained the SnRK2.6 or PYR1 orf as His_6_-thioredoxin-thrombin cleavage site-avitag fusion, and the second cloning site carried the *E. coli* biotin-ligase gene *BirA*. The 14 amino acid avitag functions as a defined in vivo biotinylation site in *E. coli*. Cells grown in the presence of 40 µM biotin were lysed and fusion proteins were purified using nickel HiTrap columns. Following the thrombin release of the His_6_-thioredoxin tag, the SnRK2.6/PYR1 proteins were loaded onto a second Ni column to separate the untagged proteins from tags. The untagged biotinylated proteins were further purified by Superdex 200 gel filtration chromatography (Amersham Biosciences) in Buffer A.

### Mutagenesis

Site directed mutagenesis was carried out using the QuikChange method (Agilent). All constructs were verified by sequence analysis.

### Phosphatase assay

The phosphatase activity of HAB1 was determined colorimetrically using a phosphatase assay kit (BioVision). 100 nM of recombinant HAB1 protein was incubated with 100 µM SnRK2.6 activation loop phosphopeptide (HSQPK (pS) TVGTP) in phosphatase assay buffer (50 mM imidazole, 5 mM MgCl_2_, 0.2 mM EGTA, pH7.2) for 30 min at room temperature. The amount of released phosphate was determined based on absorbance at 650 nm and was used to calculate the phosphatase activity of HAB1.

### Incubations with H_2_O_2_, DTT, and TCEP

Recombinant HAB1 protein was incubated with different concentrations of H_2_O_2_ for 30 min at 30°C, followed by 5 min incubation with 10 U of catalase enzyme to destroy H_2_O_2_ and the phosphatase activity was measured. Reactivation experiments after the H_2_O_2_ treatment were performed by incubating with DTT, tris (2-carboxyethyl) phosphine (TCEP), or glutathione (GSH) for 30 min at 30°C, prior to measuring the phosphatase activity.

### Kinase assays

Indicated amounts of His_6_-GST-SnRK2.6 were pre-incubated with 30 nM of the wild type or C186S/C274S mutant His_6_-MBP-HAB1(1–511 aa) protein, treated without or with H_2_O_2_ for 30 min at 30°C, in 25 mM Tris/Cl, pH 7.4, 12 mM MgCl_2_ and 0.1 mM EGTA for 30 min at room temperature. This was followed by 15 min incubation with 0.2 mM unlabeled ATP, 2.5 µCi [^32^P]-γATP, and indicated amounts of His_6_-GST-ABF2 (73–120 aa) in a total reaction volume of 15 µl. Reactions were terminated by addition of SDS sample buffer and subjected to tricine SDS-PAGE. Gels were stained with colloidal Coomassie Blue and subjected to autoradiography using a FLA-5000 phosphor imager (Fuji).

### AlphaScreen assay

Interactions between SnRK2.6(11–362 aa) or SnRK2.6 ABA box peptide and HAB1(171–511 aa) wild type, C186/274S double mutant protein and R505A mutant treated with or without H_2_O_2_ were assessed by luminescence proximity AlphaScreen assay (Perkin Elmer) as described previously[21]. All reactions contained 100 nM of biotin-SnRK2.6 or biotinylated ABA box peptide bound to streptavidin donor beads and 100 nM of His_6_-MBP-HAB1 wild type or mutant protein treated with or without H_2_O_2_ bound to nickel acceptor beads. For the interaction of PYR1 ABA receptor with HAB1, 100 nM biotin PYR1(9–182 aa) was used in the presence of 10 µM (+)-ABA.

## Results

### Reversible oxidation of HAB1 by H_2_O_2_

**Figure 1:**
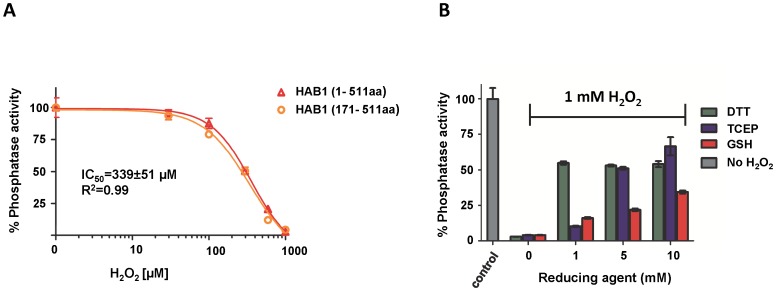
Reversible oxidation of HAB1 by H_2_O_2_. A) Sensitivity of full length (1–511 aa) and phosphatase domain (171–511 aa) of HAB1 to H_2_O_2_ treatment. B) Reactivation of H_2_O_2_ treated HAB1 by reducing agents (n = 3, error bar represent s.d.).

The ABA-signaling PP2Cs ABI1 and ABI2 have been shown to play important roles in ABA-mediated ROS signaling, and their catalytic activities are directly inhibited by H_2_O_2_ by an unknown mechanism [49], [50], [51]. During protein purification, we noticed that the activity of the related ABA-signaling PP2C HAB1 was also very sensitive to oxidizing conditions. To directly test whether HAB1's phosphatase activity is affected by H_2_O_2_ treatment, we incubated recombinant purified HAB1 protein with H_2_O_2_ and determined its ability to dephosphorylate a peptide corresponding to the phosphorylated activation loop of SnRK2.6, which is the main SnRK2 regulator of ABA signaling in guard cell and a physiological target of HAB1. On treatment with H_2_O_2_, HAB1 lost its activity with an IC_50_ of 340 µM (Fig. 1A). Both full length HAB1(1–511 aa) and the conserved class A PP2C phosphatase domain of HAB1(171–511 aa), which is sufficient and required for interactions with ABA receptors and SnRK2 kinases [21], showed very similar sensitivity to H_2_O_2_ treatment, indicating that the non-conserved N-terminus of HAB1 is not required for H_2_O_2_-mediated HAB1 inactivation. Furthermore, inactivation was partially reversible. Treatment with 1 mM H_2_O_2_ completely (>95%) abolished HAB1 phosphatase activity, while subsequent incubation with reducing agents such as DTT and TCEP restored 55 and 65% of its original activity, respectively (Fig. 1B). Treatment with reduced glutathione (GSH) was less efficient and could only restore 30% of the initial HAB1 phosphatase activity even at 10 mM concentration, similar to what had been reported for ABI2 [50].

### C186 and C274 mediate oxidative inactivation of HAB1

**Figure 2:**
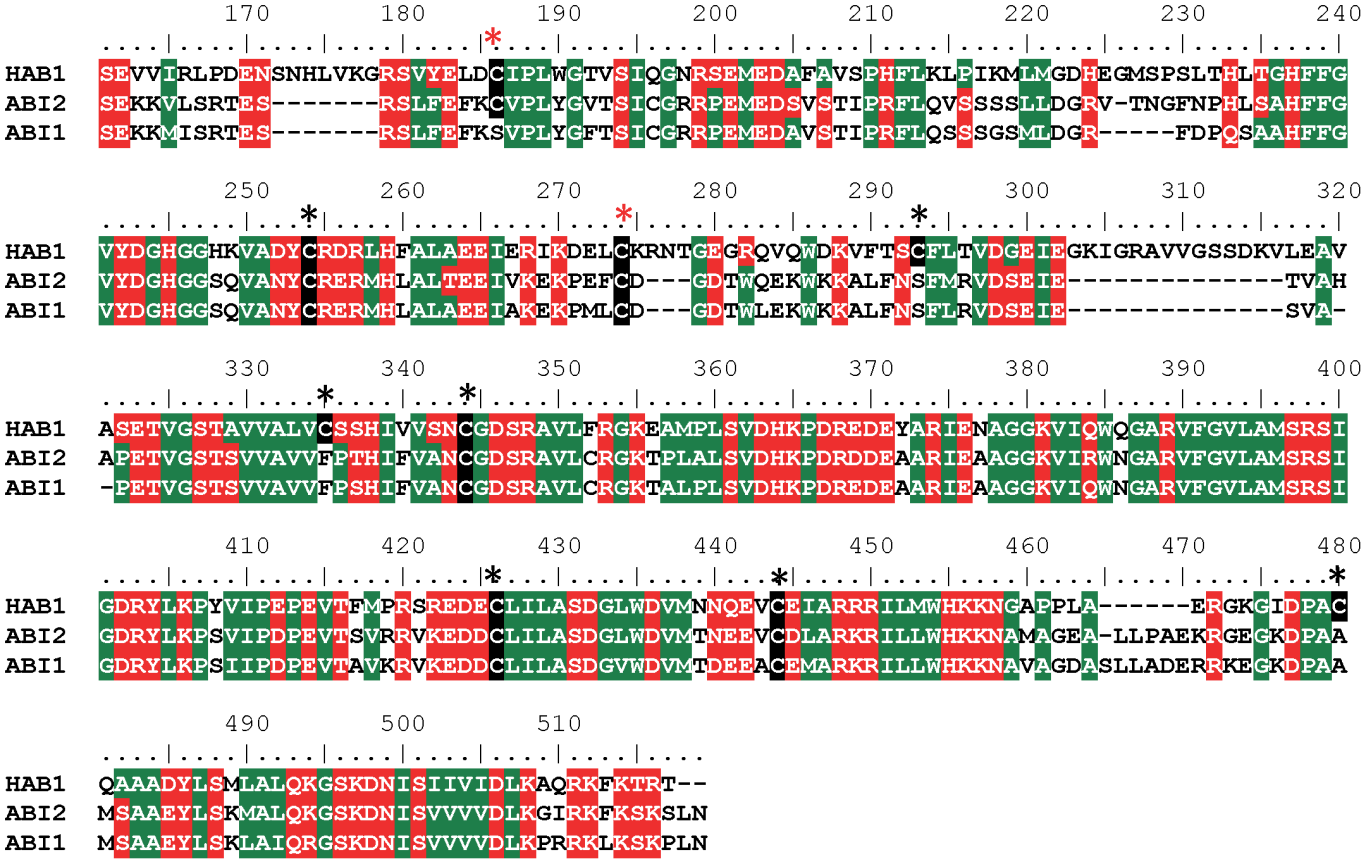
Sequence alignment of HAB1, ABI2 and ABI1. Cysteines in the phosphatase domain of HAB1 are indicated by black boxes and asterisks. C186 and C274 are highlighted by red asterisks. The numbers on top of the alignment indicate amino acid positions within HAB1.

**Figure 3:**
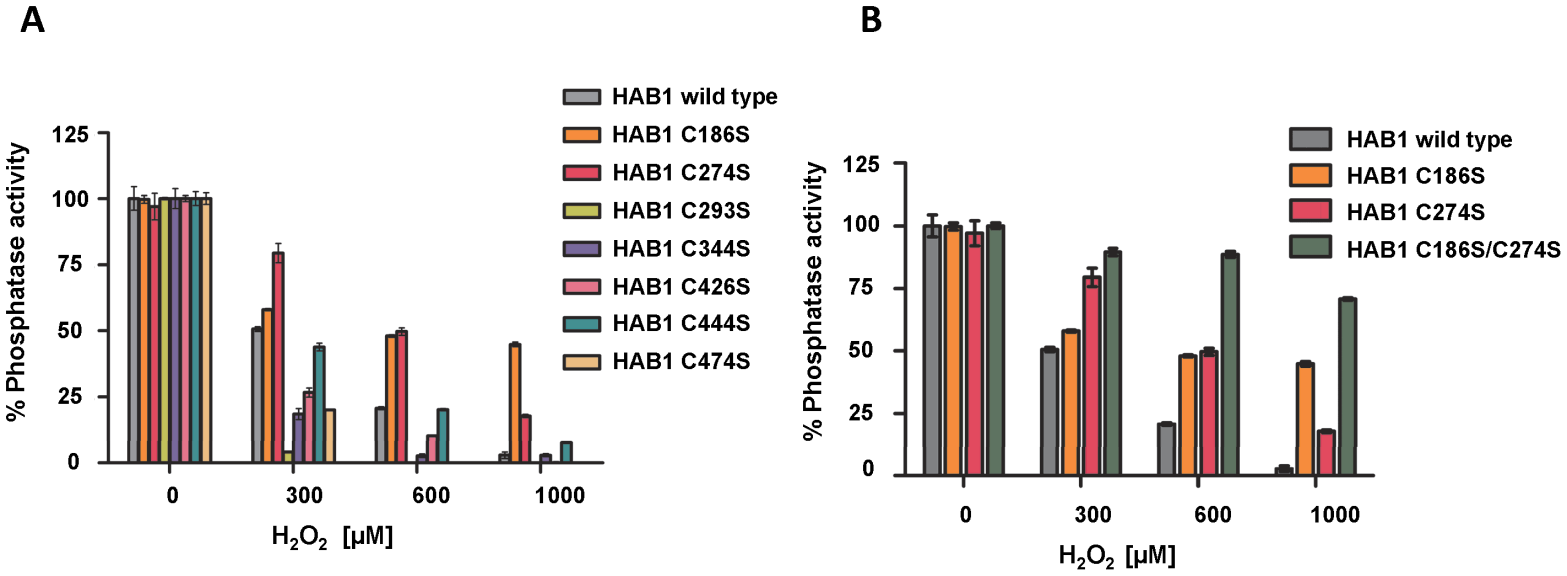
HAB1 C186S/C274S mutant protein is resistant to inactivation by H_2_O_2_. A) Relative phosphatase activities of HAB1 wild type and cysteine point mutant proteins to H_2_O_2_ treatment. B) H_2_O_2_ resistance of C186S and C274S single and double mutant proteins compared to wild type HAB1 (n = 3, error bar represent s.d.).

Since cysteines are the most common redox sensors in proteins, we first tested whether replacement of any of the nine cysteine residues in the phosphatase domain of HAB1 (Fig. 2) would affect inhibition by H_2_O_2_. The HAB1 proteins with cysteine to serine mutation of residues C254 and C335 failed to purify and hence were omitted from further analysis. The remaining seven mutant proteins were incubated with increasing concentrations of H_2_O_2_ and their phosphatase activities were determined. Two of the mutant proteins, C186S and C274S, showed significant protection against inactivation by H_2_O_2_ (Fig. 3A). Furthermore, HAB1 with C186S/C274S double mutations retained ∼70% of their phosphatase activity even after treatment with 1 mM H_2_O_2_, compared to wild type HAB1, which retained only <5% of its original activity (Fig. 3B), indicating that oxidation of both of these two cysteines contributes to HAB1 inactivation. Together, these results strongly suggest that C186 and C274 residues function as key redox sensing thiols in HAB1.

### H_2_O_2_ at moderate concentrations induces formation of catalytically inactive HAB1 dimers

**Figure 4:**
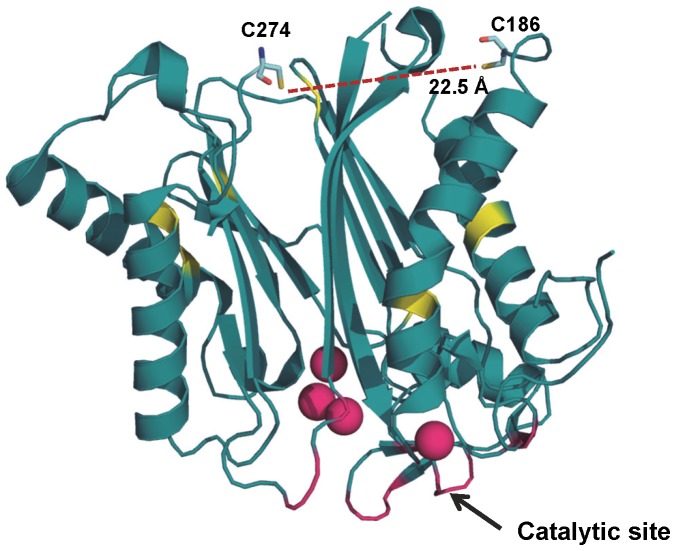
Crystal structure of HAB1. Ribbon representation of the HAB1 phosphatase domain (PDB: 4LA7) with C186 and C274 presented as stick models and the remaining cysteine residues as yellow patches. The SnRK2.6 interacting site is shown in magenta and MgCl_2_ ions as magenta spheres (figure drawn from).

**Figure 5:**
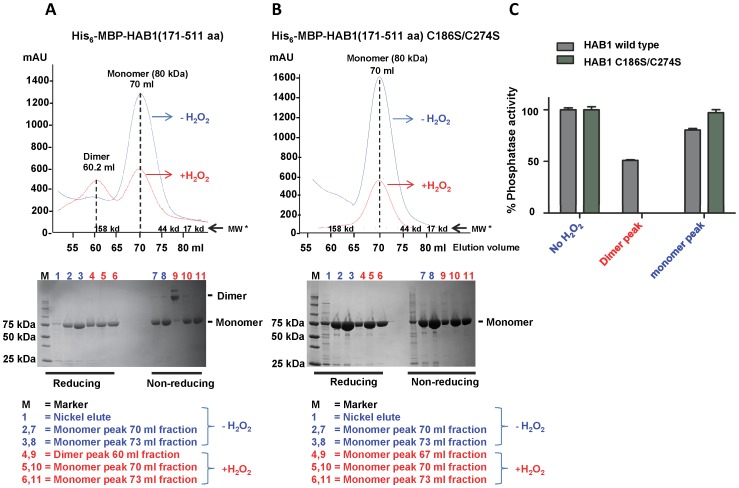
H_2_O_2_ induces formation of HAB1 dimers that are catalytically compromised. A, B, upper panels: Gel filtration profiles of HAB1 wildtype and C186S/C274S mutant protein in the presence and absence of H_2_O_2_. Wildtype His_6_-MBP-HAB1 elutes as monomers in the absence of H_2_O_2_ (blue chromatogram) and as a mixture of dimers (at 60 ml elution volume) and monomers (at 70 ml) upon treatment with 0.3 mM H_2_O_2_ (red chromatogram). In contrast, His_6_-MBP-HAB1 C186S/C274S elutes exclusively as monomers. *MW: molecular weight standards; mAU: absorbance at 280 nm×1000. Lower panels: Reducing and non-reducing SDS PAGE of gel filtration fractions of wild type and C186S/C274S HAB1 proteins, respectively. C) Phosphatase activity of HAB1 protein from the untreated monomer fraction (no H_2_O_2_), from the dimer and monomer fractions of wild type HAB1 treated with 0.3 mM H_2_O_2_, and from the monomer fraction of HAB1 C186S/C274S treated with 0.3 mM H_2_O_2_.

The crystal structure of HAB1 illustrates that the key cysteine residues C186 and C274 are located opposite to the catalytic cleft (Fig. 4), excluding the possibility that they are directly involved in catalytic activity [19]. Moreover, C186 and C274 are 22.5 Å apart from each other, and neither residue is in the vicinity of another cysteine (Fig. 4), eliminating their involvement in any intramolecular disulfide bonds in the absence of major conformational rearrangements. To test whether HAB1 oxidation leads to the formation of intermolecular disulfide bonds, we fractionated wild type and C186S/C274S mutant HAB1 phosphatase domain, either untreated or treated with 0.3 mM H_2_O_2_ for 30 min at 30°C, by size exclusion chromatography. As seen in Fig. 5A, B, both wild type and C186S/C274S double mutant His_6_-MBP-HAB1(171–511 aa) eluted as monomers in the absence of H_2_O_2_-treatment. In contrast, when treated with 0.3 mM H_2_O_2_, wild type HAB1 phosphatase domain eluted as two about equally sized peaks, corresponding to dimer and monomer fractions (Fig. 5A), while the correspondent C186S/C274S mutant proteins eluted only as monomers in the presence of 0.3 mM H_2_O_2_ (Fig. 5B). Separation of the size exclusion chromatography fractions by reducing and non-reducing SDS PAGE (Figs. 5A, B bottom panels) demonstrated that H_2_O_2_-treated wild type HAB1 from dimer fractions migrated as dimers under non-reducing conditions and as monomers under reducing conditions, indicating that dimers are formed by intermolecular disulfide bonding. When we tested the fractions containing the untreated (monomer) wild type and mutant HAB1 proteins as well as the H_2_O_2_-treated wild type (monomer and dimer) and mutant (monomer) fractions, we found that dimeric HAB1 lost >50% of its phosphatase activity while monomeric HAB1 had the same or only slightly reduced phosphatase activity compared to untreated protein (Fig. 5C). Together, these results strongly suggest that the partial loss of HAB1 phosphatase activity upon treatment with 0.3 mM H_2_O_2_ is due to formation of catalytically compromised HAB1 generated by the formation of intermolecular disulfide bonds involving cysteines C186 and C274.

### H_2_O_2_ reversibly inactivates the ability of wild type HAB1, but not HAB1 C186S/C274S, to inhibit SnRK2.6 kinase activity

**Figure 6:**
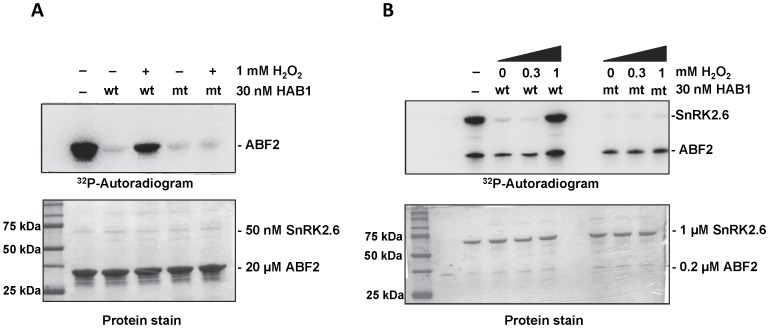
H_2_O_2_ prevents the HAB1-mediated inhibition of SnRK2.6 kinase activity. A) SnRK2.6 trans-phosphorylation activity at high (30∶50 molar) HAB1:SnRK2.6 ratio. B) SnRK2.6 auto- and trans-phosphorylation activity at low (30∶1000 molar) HAB1:SnRK2.6 ratio. Recombinant SnRK2.6 was incubated at the indicated concentrations with a fragment of the transcription factor ABF2 [GST-ABF2(73–120 aa)] and with ^32^P-γATP in the presence and absence of H_2_O_2_-treated wild type (wt) and mutant (mt; C186S/C274S) HAB1.

In the absence of ABA, HAB1 binds to ABA-signaling SnRK2s and inhibits their kinase activity. In the presence of ABA, HAB1 forms complexes with PYR/PYL/RCAR receptors that keep HAB1 in an inactive form, thereby relieving inhibition of SnRK2s and allowing SnRK2s to phosphorylate their downstream targets. To test whether H_2_O_2_ treatment could also relieve PP2C-mediated SnRK2 inhibition, we performed radioactive kinase assays using purified SnRK2.6/OST1, the key SnRK2 protein in guard cells, in the presence and absence of untreated and H_2_O_2_-treated HAB1. At a close to 1∶1 HAB1:SnRK2.6 stoichiometric ratio, HAB1 strongly inhibited the phosphorylation of a fragment from ABF2, a physiological substrate of SnRK2.6, as previously reported [21] (Fig. 6A). Even in the presence of a 30-fold higher concentration of SnRK2.6 than HAB1, HAB1 still effectively inhibited SnRK2.6 autophosphorylation, which is more sensitive to inhibition than transphosphorylation [21] (Fig. 6B). At this ratio, HAB1 partially inhibited transphosphorylation of ABF2 [21] (Fig. 6B), consistent with the requirement of 1∶1 HAB1:SnRK2.6 complex formation for full inhibition of SnRK2.6 kinase activity. Similar to ABA, treatment with 1 mM H_2_O_2_, which inhibits >95% HAB1 phosphatase activity, completely abolished the ability of HAB1 to inhibit SnRK2.6 auto- and transphosphorylation activity at substoichiometric levels (Fig 6B), and strongly compromised its ability to inhibit SnRK2.6 transphosphorylation at near stoichiometric levels (Fig. 6A). Importantly, the HAB1 C186S/C274S mutant continued to exert inhibition on SnRK2.6 and thereby prevented both auto-phosphorylation of SnRK2.6 and transphosphorylation of ABF2 even after treatment with 1 mM H_2_O_2_ (Figs. 6A, B). Thus our kinase assay results show that H_2_O_2_ treatment can lead to inhibition of HAB1, thereby allowing SnRK2.6 autoactivation and substrate phosphorylation. Together, these results indicate that H_2_O_2_ at high concentrations can inhibit HAB1 to activate SnRK2.6 kinase in the absence of ABA-bound PYR/PYL/RCAR receptors and that this inhibition requires HAB1 C186 and C274 as oxidation-sensitive thiols.

### H_2_O_2_ inhibits direct interaction of HAB1 with SnRK2.6

**Figure 7:**
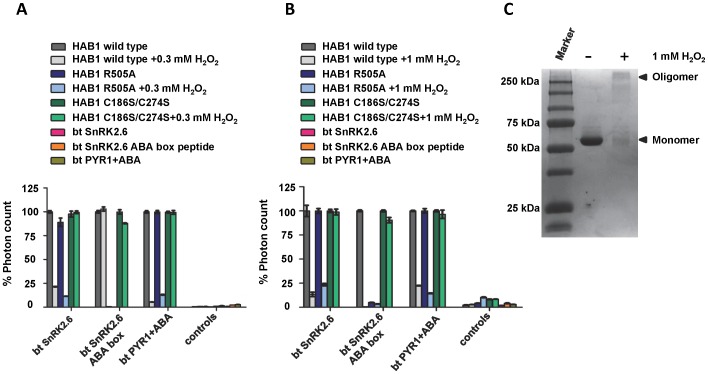
H_2_O_2_ inhibits the direct interaction of HAB1 with SnRK2.6 and PYR1. A, B) AlphaScreen assays showing interactions between His_6_-MBP-HAB1(171–511 aa) wild type, C186/274S double mutant and R505A mutant protein and biotin-SnRK2.6(11–362 aa), biotin-SnRK2.6 ABA box peptide and biotin-PYR1(9–182 aa) with 10 µM ABA in the absence or presence of 0.3 mM (A) or 1 mM H_2_O_2_ (B). Controls are HAB1 proteins in the absence of SnRK2.6 or PYR1 as well as SnR2.6 or PYR1 in the absence of HAB1 (n = 3, error bar represent s.d.). C) Non-reducing SDS PAGE of untreated wild type HAB1 (−) and HAB1 treated with 1 mM of H_2_O_2_ (+).

The crystal structure of the SnRK2.6/HAB1 complex revealed that the catalytic clefts of the SnRK2.6 kinase domain and the HAB1 phosphatase domain directly interact with each other [21]. This packing enables HAB1 to inhibit SnRK2.6 kinase activity by two different mechanisms: catalytically, by dephosphorylating S175 of the kinase activation loop, which inserts deeply into the HAB1 catalytic cleft, thereby reducing SnRK2.6 kinase activity, and stoichiometrically, by forming a complex in which HAB1 sterically blocks SnRK2.6 substrate access and keeps the kinase domain in an inactive and wide-open conformation. The latter mechanism completely abolishes kinase activity of SnRK2.6/HAB1 complexes, even when complexes are formed with catalytically inactive HAB1 mutant protein [21]. We therefore wanted to test if the H_2_O_2_ treatment of HAB1 would also affect its stable physical interaction with SnRK2.6, thereby blocking both mechanisms of SnRK2.6 inhibition. Using an AlphaScreen luminescence proximity assay, we could detect a clear interaction between biotinylated SnRK2.6 and His_6_-MBP-tagged wild type and C186S/C274S HAB1 (Fig. 7). Treatment with either 0.3 mM (Fig. 7A) or 1 mM (Fig. 7B) H_2_O_2_ strongly reduced the interaction with wild type HAB1, but had no effect on the interaction with HAB1 mutant protein. This demonstrates that H_2_O_2_ treatment indeed inhibits both HAB1 catalytic activity, required to reduce SnRK2.6 kinase activity at low PP2C levels, and stable SnRK2.6–HAB1 interaction, required for full kinase inactivation at high PP2C levels. On the other hand, HAB1 C186S/C274S could interact with SnRK2.6 irrespective of its treatment with H_2_O_2_, further supporting that these two cysteines are critical for redox sensing.

HAB1 and SnRK2.6 interact with each other via two discrete interaction surfaces: the first interaction interface is formed by the catalytic sites of SnRK2.6 and HAB1, and that interaction is required for inhibition of SnRK2.6 by HAB1. The second interface is between the flexible C-terminal ABA box motif of SnRK2.6 and the back side of HAB1, which stabilizes the interaction without directly contributing to inhibition of the kinase activity. Treatment with 0.3 mM H_2_O_2_ had no effect on the specific interaction between HAB1 and the SnRK2.6 ABA box (Fig. 7A), further demonstrating that this treatment does not simply lead to denaturation or gross misfolding of wild type HAB1, and indicating that inhibition of the SnRK2.6-HAB1 interaction is likely due to the reduced ability of SnRK2.6 to interact with the catalytic site of H_2_O_2_–treated HAB1. In contrast, treatment of HAB1 with 1 mM H_2_O_2_ led to the formation of higher molecular weight oligomer species (Fig. 7C) and to the loss of the interaction between HAB1 and ABA box peptide (Fig. 7B). We cross-validated the selective inhibition of only the HAB1–SnRK2.6 interaction of the catalytic sites at 0.3 mM H_2_O_2_ using the His_6_-MBP-HAB1 R505A mutant protein, which is incapable of binding to the SnRK2.6 ABA box peptide and hence can only interact with SnRK2.6 via the catalytic site interaction [21]. HAB1 R505A mutant protein behaved very similar to wild type HAB1, confirming that only the catalytic site interaction is effected by treatment with 0.3 mM H_2_O_2_ (Fig 7A, B).

Given that treatment with moderate concentrations of H_2_O_2_ appears to affect both activities at the catalytic site (phosphatase activity and SnRK2.6 interaction), we reasoned that oxidation likely modulates this site. The “gate” loop of the PYR/PYL/RCAR receptors also binds the catalytic cleft of HAB1, mimicking the activation loop of SnRK2.6, and receptors and SnRK2 kinases compete for binding to the HAB1 catalytic site. We therefore tested whether the H_2_O_2_ treatment of HAB1 also affected the ABA-inducible interaction with the PYR1 receptor in the presence of ABA. As shown in Figs. 7A and B, the H_2_O_2_ treatment reduced the binding signal of wild type HAB1, but not HAB1 C186S/C242S, by more than 70%, consistent with significant H_2_O_2_-mediated changes at the HAB1 catalytic site.

## Discussion

In our present study we show that HAB1, like ABI1 and ABI2, is sensitive to H_2_O_2_ treatment. This result was expected since all three PP2Cs are functionally redundant and are co-localized in guard cells, suggesting that they need to be collectively inhibited to prevent SnRK2.6 inhibition in vivo. Although HAB1 is less sensitive to H_2_O_2_ when compared to ABI1 and ABI2, the experimental findings from Meinhard et al. indicate 0.1 to 0.3 mM as the intracellular resting levels of H_2_O_2_ in *Arabidopsis* without taking into account the subcellular compartmentalization [50]. There may also be a localized increase in H_2_O_2_ levels during stress. These levels are further consistent with the ones determined in tobacco leaves, suggesting that oxidation of HAB1 by H_2_O_2_ is physiological at an IC_50_ of about 0.3 mM [52]. A partial inactivation of HAB1 at these levels could also play a major role in activating downstream signaling of the ABA pathway. Micromolar levels of ABA induce H_2_O_2_ production in guard cells by activating NADPH oxidases. In turn, H_2_O_2_ can inactivate PP2Cs, the major negative regulators of ABA signaling, suggesting that regulation of PP2Cs by H_2_O_2_ may act as a feedforward mechanism to amplify ABA downstream signaling. PP2C inhibition allows activation of SnRK2.6, a major limiting factor of the ABA signaling pathway leading to the closure of stomatal pores during water stress. On the other hand, inactivation of HAB1 by H_2_O_2_ is partially reversible. Our reactivation assays with DTT and TCEP could restore 55–65% of the initial activity of HAB1. HAB1 treated with 1 mM H_2_O_2_ led to the formation of higher order oligomers (Fig. 7C), suggesting that at this concentration outside of a cellular environment some protein molecules might have been denatured. This could be the reason why 100% activity could not be restored using reducing agents. Our findings suggest that H_2_O_2_ generated during water stress can oxidize and inactivate HAB1, while upon stress relief HAB1 could be reactivated by cellular reducing thiols allowing it to inhibit SnRK2.6 and terminate downstream signaling.

Full length HAB1 and its phosphatase domain alone showed similar sensitivity to H_2_O_2_, suggesting that the phosphatase domain is sufficient for oxidative inactivation. We replaced each of the nine cysteines of the phosphatase domain with serine and identified C186 and C274 as the key residues that are susceptible for oxidative inactivation by H_2_O_2_, as the C186S/C274S HAB1 double mutant was almost completely insensitive to H_2_O_2_ treatment. This is consistent with PAO inhibition studies on ABI1 and ABI2, which also suggest cysteine residue(s) as probable candidates for oxidation by H_2_O_2_ [49]. The requirement of C186 and C274 for the inhibition of both phosphatase activity and kinase binding further supports their role as redox sensing thiols in HAB1.

Protein phosphatases have been one of the major targets of H_2_O_2_-mediated oxidation [53]. Many phosphatases have nucleophilic cysteine residues in their catalytic site that upon oxidation form inter/intra molecular disulfide bonds, sulfenamide intermediates, or glutathione conjugates to inactivate their phosphatase activities [54], [55], [56], [57], [58]. However, C186 and C274 are in a distal position from the HAB1 catalytic site (Fig. 4), excluding the possibility that these two residues could be directly involved in either catalysis or in the SnRK2.6 interaction mediated by the catalytic site interface. Incubation of wild type HAB1, but not HAB1 C186S/C174S, with 0.3 mM H_2_O_2_ induced formation of dimers that were fully convertible to monomers under reducing conditions (Fig. 5). In contrast to the monomeric fractions, HAB1 from the dimeric fraction was compromised both catalytically and in physical interaction with its main downstream target, SnRK2.6, and its upstream regulator PYR1. Collectively, these results suggest that moderate concentrations of HAB1 lead to the formation of dimers by intermolecular disulfide bonds that involve C186 and C274. The position of these two cysteines distal to the HAB1 catalytic site indicates that dimer formation would not restrict substrate access to the catalytic cleft. Though our experimental results do not directly determine the mechanism, they suggest that reversible dimerization by the oxidation of C186 and C274 may cause conformational changes that in turn lead to an altered catalytic cleft. Conformational changes by oxidation-induced formation of a disulfide bond between two distant cysteine residues was first observed for the bacterial redox sensor OxyR [59].

**Figure 8:**
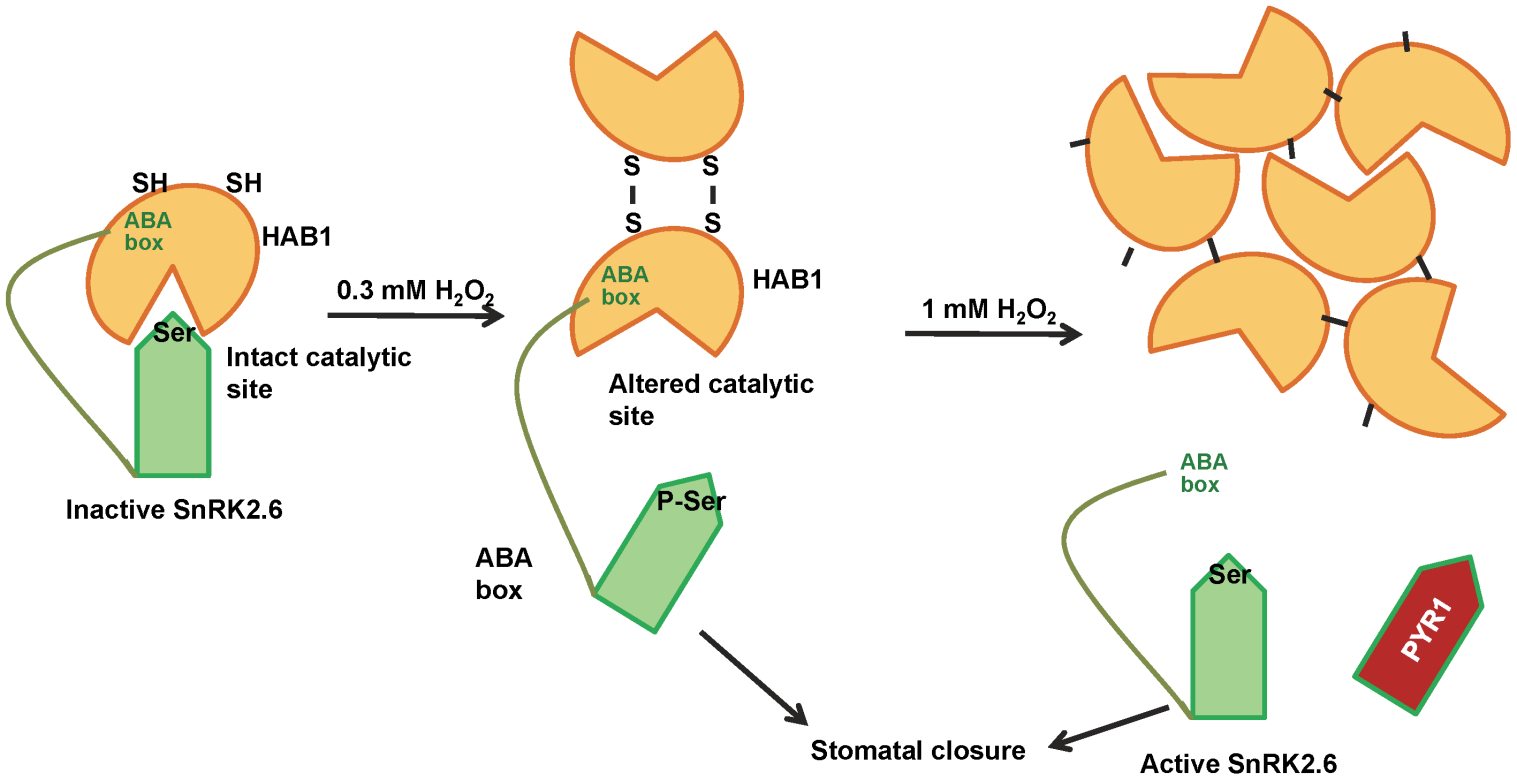
Cartoon representation of the regulation of HAB1 by H_2_O_2_ during water stress. See text for details.

Based on our results we therefore propose a model to explain the mechanism by which H_2_O_2_ regulates HAB1 (Fig. 8). Oxidation of C186 and C274 at moderate H_2_O_2_ levels leads to dimerization by intermolecular disulfide bond formation. This dimer species is associated with changes at the catalytic site that partially inactivate the enzymatic activity of HAB1 and block the interaction of the SnRK2.6 kinase with the catalytic site, but not binding of the SnRK2.6 ABA box to the back site of HAB1. At high H_2_O_2_ concentrations, HAB1 forms oligomeric species that are both catalytically inactive and incapable of binding interaction partners at either side of HAB1, suggesting that the oligomeric species may be largely inaccessible to both interaction partners and possibly also to peptide substrate. The combined effect of the inhibition of catalytic activity and substrate binding can lead to auto-activation of SnRK2.6 allowing it to phosphorylate its downstream substrates to mediate stomatal closure during water stress. Hence, HAB1 can act as a redox-sensing switch, supporting that H_2_O_2_-mediated PP2C inactivation may play a major role in regulation of ABA signaling during water stress.
